# Impact of postsurgical vaginal microbiome on high-risk HPV infection and recurrence risk in patients with cervical cancer and intraepithelial neoplasia: A retrospective study

**DOI:** 10.1016/j.gore.2024.101506

**Published:** 2024-09-11

**Authors:** Yan Ma, Lijuan Wan, Ruonan Li, Xixi Chen, Huiyan Wang

**Affiliations:** a Department of Gynecologic Oncology, The First Affiliated Hospital of USTC, Division of Life Sciences and Medicine, University of Science and Technology of China, Hefei, Anhui 230001, PR China; b Department of Laboratory Diagnostics, The First Affiliated Hospital of USTC, Division of Life Sciences and Medicine, University of Science and Technology of China, Hefei, Anhui 230001, PR China; c Department of Obstetrics and Gynecology, The First Affiliated Hospital of Chongqing Medical University, Chongqing 400016, PR China

**Keywords:** Vaginal microbiome, Cervical cancer, Cervical intraepithelial neoplasia, Surgical treatment, Disease recurrence, Lactobacilli

## Abstract

**Purpose:**

This study aimed to determine the effect of postsurgical vaginal microbiome (VM) on high-risk human papillomavirus (hrHPV) infection and the risk of disease recurrence in patients surgically treated for cervical cancer (CC) or intraepithelial neoplasia (CIN).

**Methods:**

207 women who underwent surgical treatment for CC or CIN at the Department of Gynecologic Oncology of the First Affiliated Hospital of University of Science and Technology of China from November 2016 to October 2023 were included. The patients’ clinical data, including age, surgical modality, and diagnosis at time of index surgery, were collected retrospectively and analyzed. Associations between postsurgical VM indices, hrHPV infection, cervical cytology, and recurrence were also evaluated.

**Results:**

Patient age, surgical modality (whether complete excision of the cervix was performed), and diagnosis at time of index surgery (cervical dysplasia vs. cervical carcinoma) showed no significant association with postsurgical hrHPV infection, cervical cytology, or disease recurrence. However, postsurgical VM imbalance was significantly associated with hrHPV infection status (OR = 4.640, 95 % CI = 2.085–10.460, *P* < 0.001), abnormal cervical cytology (OR = 3.994, 95 % CI = 1.154–13.826, *P* = 0.020), and disease recurrence (OR = 3.789, 95 % CI = 1.091–13.154, *P* = 0.026). Among the specific VM indices, a vaginal pH above 4.5 (OR = 4.570, 95 % CI = 1.640–12.690, *P* = 0.002), a lactobacilli proportion below 50 % (OR = 3.938, 95 % CI = 1.299–11.934, *P* = 0.010), and the presence of aerobic vaginitis (AV, OR = 2.425, 95 % CI = 0.996–5.901, *P* = 0.046) were risk factors for postsurgical recurrence.

**Conclusion:**

Postsurgical VM imbalance, especially abnormal indices, such as a pH above 4.5, a lactobacilli proportion below 50 %, and the presence of AV, was associated with an increased risk of postsurgical recurrence in patients who underwent surgical treatment for CIN and CC. Monitoring and potentially intervening in the VM may improve the prognosis of these patients.

## Highlights

• Postsurgical VM imbalance indicates an increased risk of hrHPV infection in CIN/CC patients.
• Postsurgical VM imbalance was also associated with cervical cytology abnormality, and CIN/CC recurrence.
• Vaginal pH and lactobacilli proportion are specific postsurgical VM indices.

## Graphical abstract

**Figure:**
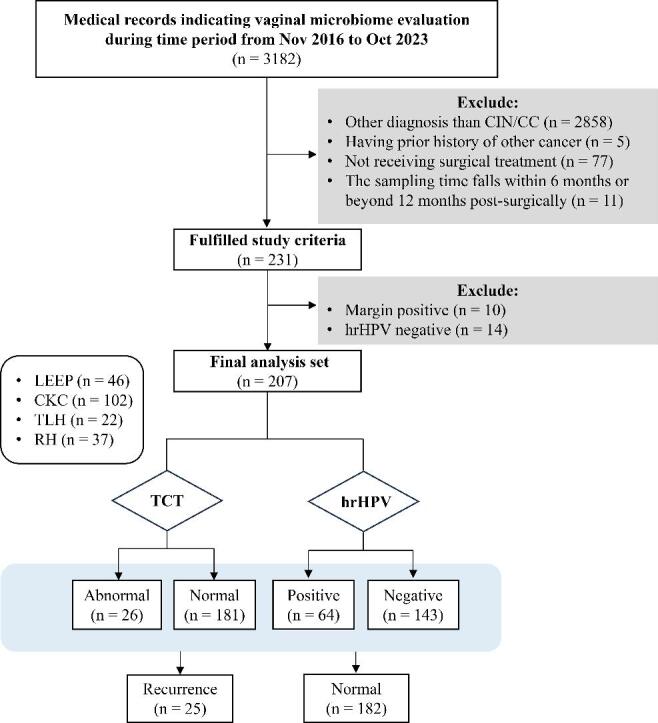

## 1 Introduction

Cervical cancer (CC) is one of the most common malignancies affecting women globally (Bray et al., 2024). In 2022, CC accounted for approximately 660,000 new cases and 350,000 mortalities worldwide (Bray et al., 2024). Persistent infection with high-risk human papillomavirus (hrHPV) has been established as the primary etiological factor for cervical intraepithelial neoplasia (CIN) and the subsequent development of CC (Ferrall et al., 2021). Alterations in the vaginal microbiome (VM) are known to play a role in the onset and progression of CIN and CC (Wang et al., 2019). Furthermore, a decrease in lactobacilli populations and increased bacterial diversity have been associated with hrHPV infection and the development of CIN (Fang et al., 2022, Liu et al., 2022).

Surgical resection is the cornerstone of CIN and CC management (Brucker and Ulrich, 2016, Shastri et al., 2022), as it significantly diminishes the likelihood of disease progression and recurrence (Khunamornpong et al., 2015). However, compared with the general population, women who have undergone surgical treatment have a two- to fivefold greater risk of recurrence or progression to invasive carcinoma (Strander et al., 2007, Katki et al., 2013). Persistent and new-onset hrHPV infections are major contributors to postsurgical recurrence in patients with CIN or CC (Andrade et al., 2014, Bogani et al., 2020). Although VM is closely associated with hrHPV infection and the progression of CIN in individuals who have not undergone surgery, limited research has been conducted on postsurgical VM in patients treated for CIN or CC (Caselli et al., 2020). Consequently, it remains unclear whether the postsurgical VM influences hrHPV infection and disease recurrence in these patients. This study aimed to elucidate the influence of postsurgical VM on hrHPV infection and disease recurrence in patients who were treated surgically for CIN or CC. The findings are anticipated to enhance understanding and guide both monitoring and intervention strategies.

## 2 Methods

### 2.1 Study population

Clinical data were retrospectively collected from the electronic medical records of 3182 women who underwent VM examinations at the Department of Gynecologic Oncology of the First Affiliated Hospital of University of Science and Technology of China between November 2016 and October 2023. The eligibility criteria were that the participants were female patients who had been diagnosed with CIN or CC and subsequently underwent surgical intervention, including loop electrosurgical excision procedure (LEEP), cold knife conization (CKC), laparoscopic total hysterectomy (TLH), and radical hysterectomy (RH), in the past 6–12 months. Furthermore, eligible participants were required to have no prior history of cancer, not be pregnant or breastfeeding, and test negative for human immunodeficiency virus (HIV). The exclusion criteria included a negative hrHPV test result at the time of surgery or a positive surgical margin postintervention.

### 2.2 Sample collection

Cervical specimens were collected for hrHPV detection and the ThinPrep cytologic test (TCT). Vaginal swabs were also collected alongside cervical specimens to assess the VM indices. The Tongren Medical Digital Electronic Colposcope System (Tongren, China) was used to perform colposcopic examinations and to obtain biopsy samples.

### 2.3 hrHPV examination

The hrHPV examination was performed via a Zhongsheng Fangzheng Human Papillomavirus Genotyping Kit (Zhongsheng Fangzheng, China). A Light Cycler LC480 PCR instrument (Roche, Germany) was used to detect fifteen hrHPV subtypes: 16, 18, 31, 33, 35, 39, 45, 51, 52, 53, 56, 58, 59, 66, and 68.

### 2.4 Cervical cytology and histology

The cervical cytological and histological analyses were completed by the pathology department of the hospital. Following the preparation of liquid-based thin-layer slides, the cervical specimens were diagnosed by cytologists via the TBS (the Bethesda system) classification system (Nayar and Wilbur, 2015). The pathological specimens were evaluated by at least two expert pathologists. Disease recurrence was defined as the reappearance of cervical dysplasia or cancerous tissue at the cervix or at the cervical stump.

### 2.5 VM assessment

Vaginal secretions were collected using two sterile cotton swabs for VM analyses. For direct examination, one swab was smeared onto a glass slide, subjected to Gram staining, and examined under an oil immersion microscope (Olympus, Japan). The second swab was placed into a tube, to which 8–10 drops of saline were added. After sufficient squeezing, the secretions were washed, and the swabs were removed. A 50-µL aliquot of the eluate was dropped onto a glass slide, covered with a cover slip, and left to stand for 3–5 min before microscopy was performed. The morphological microscopy examination included leukocytes, epithelial cells, dominant bacteria, trichomonas, fungal hyphae, and spores. On the basis of the microscopy results, analyses of bacterial density, flora diversity, Nugent score, and Donders score were conducted (Nugent et al., 1991, Donders et al., 2011, Yue et al., 2015, Huang et al., 2021). Furthermore, an additional 50 µL of eluate was subjected to a functional assay using the Shidasi Vaginal Microbiome Test Kit (Shidasi, China) to assess parameters such as pH, hydrogen peroxide (H_2_O_2_) production, leukocyte esterase activity, and sialidase activity (Chen et al., 2024).

The bacterial density refers to the degree of distribution of bacteria in a sample, which can reflect the total biomass of the microbiome. The classification of bacterial density is based on the average number of bacteria per field of view under each oil immersion lens: 1–9 bacteria per field are defined as Grade I, 10–99 bacteria as Grade II, 100–999 bacteria as Grade III, and >1000 bacteria as Grade IV. Flora diversity is graded on the basis of the average number of bacterial species per field of view under the oil immersion lens, reflecting the complexity of bacterial species distribution in the sample. The identification of 1–3 bacterial species per field was defined as Grade I, 4–6 species as Grade II, 7–9 species as Grade III, and >10 species as Grade IV. The presence of bacterial vaginosis (BV) was diagnosed with a Nugent score of 7 or higher (Nugent et al., 1991), and the presence of aerobic vaginitis (AV) was indicated by a Donders score of 3 or higher (Donders et al., 2001). The balance in VM was defined as Grade II–III bacterial density and flora diversity, a vaginal pH < 4.5, a leukocyte count of <10 per high-power microscopic field, and lactobacilli as the dominant bacteria identified. If any one of the bacterial density, flora diversity, leukocyte count, dominant bacteria, or pH value was abnormal, this status was defined as VM imbalance (Huang et al., 2021).

### 2.6 Statistical analysis

Statistical analysis was performed via SPSS version 21.0 (IBM Corp., Armonk, NY, USA). The skewed distribution quantitative data were represented by median and inter-quartile range, and differences between two groups were detected by non-parametric test. The differences in qualitative data between groups were determined via the chi-square test, and ranked data from different groups were analyzed via the Mann-Whitney *U* test. The odds ratios (ORs) and 95 % confidence intervals (CIs) were analyzed via the chi-square test. All the statistical tests were two-sided and considered to be significant at *P* < 0.05.

## 3 Results

**Fig. 1:**
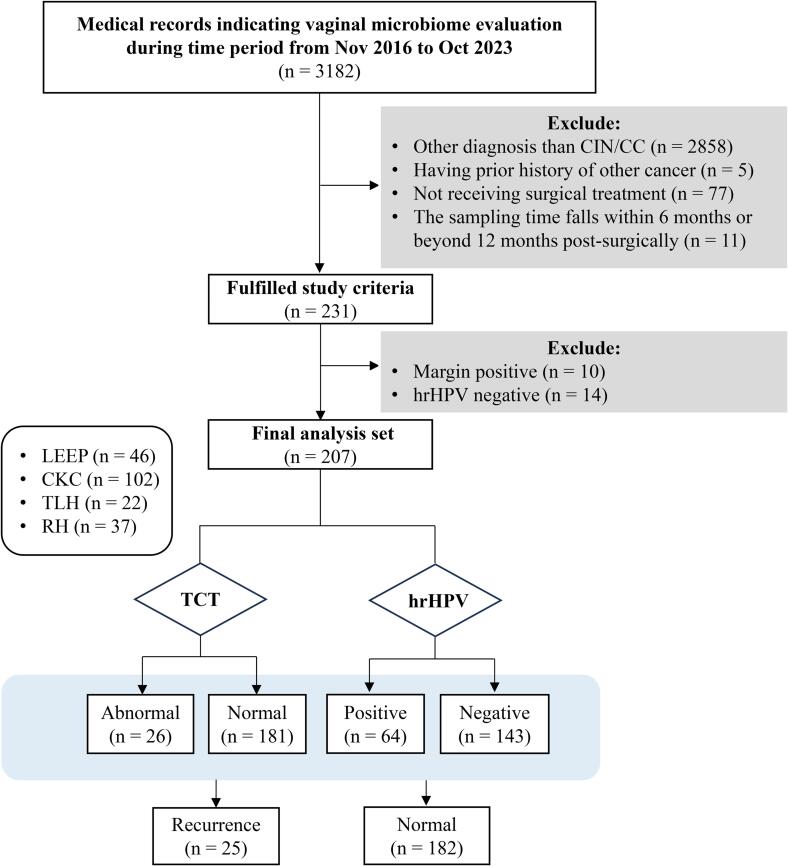
Study groups and analytical flow diagram.

After the clinical data of the 3182 women who underwent VM examinations at the First Affiliated Hospital of USTC between November 2016 and October 2023 were screened, 231 women met the eligibility criteria. Fourteen women who had negative hrHPV results at the time of surgery, along with 10 women with positive surgical margins postintervention, were excluded. Consequently, a cohort of 207 women was included in the analysis (Fig. 1). The median age (interquartile range, IQR) of the cohort was 46.0 (38.0–51.0) years, and 55 of the 207 patients (26.6 %) were postmenopausal. Among the 207 patients included in this study, 59 underwent hysterectomy (22 TLH and 37 RH) with complete excision of the cervix, whereas the other 148 underwent LEEP or CKC, by which the cervix was not excised completely.

Histological analysis of surgical specimens revealed CIN1 in 11 patients, CIN2 in 40 patients, CIN3 in 112 patients, and cervical carcinoma in 44 patients (39 squamous-cell carcinomas, 4 adenocarcinomas, and 1 adenosquamous cell carcinoma).

At 6–12 months after surgery, hrHPV was detected in 64 of the 207 patients, whereas 143 tested negative for hrHPV. Cervical cytology yielded normal results in 181 patients but revealed abnormalities in 26 patients. Upon review of all colposcopy results for patients with positive hrHPV or abnormal TCT results, histopathological examination of cervical biopsy tissues revealed pathological abnormalities suggestive of recurrence in 25 patients. Specifically, there were 21 cases of CIN1, 2 cases of CIN2, and 2 cases of CIN3. The detailed patient information is presented in Table 1 and Supplementary Table 1.

**Table 1:** Characteristics of the 207 patients included in this study.

Characteristics	Total (n = 207)
Age, median (IQR), y	46.0 (38.0, 51.0)

Menopause	
Pre	152
Post	55

Surgical modality	
LEEP	46
CKC	102
TLH	22
RH	37

Diagnosis at time of index surgery	
CIN1	11
CIN2	40
CIN3	112
Carcinoma	44

hrHPV status	
Negative	143
Positive	64

Cervical cytology	
Normal	181
Abnormal	26

Recurrence	
No	182
Yes	25
VM	
Balance	65
Imbalance	142

Bacterial density	
I	21
II–III	120
IV	66

Flora diversity	
I	12
II–III	169
IV	26

Lactobacilli proportion	
<50 %	125
≥50 %	82

Leukocyte	
<10/HF	137
≥10/HF	70

Pathogen	
Spore/hyphae	16
Trichomonad	3

BV	
Absence	181
Presence	26

AV	
Absence	105
Presence	102

pH	
<4.5	102
≥4.5	105

Leukocyte esterase	
Negative	116
Positive	91

The 207 women included were grouped according to their postsurgical hrHPV, cervical cytology, and recurrence status. The patients’ characteristics were comparatively evaluated. As indicated in Table 2, variables such as age, surgical modality, and diagnosis at time of index surgery did not significantly differ between patients who tested positive and those who tested negative for hrHPV. However, the hrHPV-positive group presented a greater incidence of VM imbalance, abnormal cervical cytology, and disease recurrence than did the hrHPV-negative group (*P* < 0.05). Similarly, there were no significant differences in variables, such as age, surgical modality, or diagnosis at time of index surgery, between patients with normal and abnormal cervical cytology results. The group with abnormal cervical cytology presented a greater prevalence of VM imbalance, hrHPV positivity, and disease recurrence. No differences were observed in surgical modality or diagnosis at time of index surgery between the recurrence group and the nonrecurrence group. However, patients in the recurrence group were older, had a greater proportion of VM imbalance, and presented higher rates of hrHPV positivity and cytological abnormalities. These findings suggest a significant association between VM, hrHPV, cervical cytology, and disease recurrence in postsurgical patients. Regardless of the chosen endpoint for analysis, VM is clearly associated with postsurgical patients' diseases. Therefore, a detailed analysis of the individual indices of VM and their interrelationships was conducted.

**Table 2:** Associations between patient characteristics, postsurgical hrHPV status, cervical cytology, and disease recurrence.

Characteristics	hrHPV			Cervical cytology			Recurrence		
	Negative (n = 143)	Positive (n = 64)	P	Normal (n = 181)	Abnormal (n = 26)	P	No (n = 182)	Yes (n = 25)	P
Age, years									
median (IQR)	45.0 (37.0–50.0)	46.0 (38.3–52.8)	0.259	45.0 (38.0–51.0)	37.5 (38.3–52.0)	0.649	45.0 (37.8–51.0)	49.0 (43.0–57.0)	**0.020**

Surgical modality									
LEEP/CKC	101	47	0.679	132	16	0.229	134	14	0.067
TLH/RH	42	17		49	10		48	11	

Diagnosis at time of index surgery									
Cervical dysplasia	110	53	0.338	144	19	0.450	146	17	0.161
Cervical carcinoma	33	11		37	7		36	8	

VM									
Balance	57	8	**<0.001**	62	3	**0.02**	62	3	**0.026**
Imbalance	86	56		119	23		120	22	

hrHPV									
Negative	143	0	−	135	8	**<0.001**	139	4	**<0.001**
Type 16/18	0	12		8	4		5	7	
Other high-risk	0	52		38	14		38	14	

Cervical cytology									
Normal	135	46	**<0.001**	181	0	−	170	11	**<0.001**
Abnormal	8	18		0	26		12	14	

Recurrence									
No	139	43	**<0.001**	170	12	**<0.001**	182	0	−
Yes	4	21		11	14		0	25	

As shown in Table 3, no correlation was found between bacterial density, flora diversity, or the presence of BV and postsurgical hrHPV, cervical cytology, or disease recurrence; similarly, neither the leukocyte count nor the presence of leukocyte esterase was associated with these three conditions. Conversely, a significant correlation was observed between the vaginal pH and lactobacilli proportion with hrHPV, cervical cytology, and recurrence. Specifically, patients with a greater vaginal pH (above 4.5) presented significantly higher rates of hrHPV infection (45.7 % vs. 15.7 %, *P* < 0.001), abnormal cervical cytology (20.0 % vs. 4.9 %, *P* = 0.001), and recurrence (19.0 % vs. 4.9 %, *P* = 0.002) than patients with a lower vaginal pH. Patients with a greater proportion of lactobacilli (above 50 %) had significantly lower incidences of hrHPV infection (14.6 % vs. 41.6 %, *P* < 0.001), abnormal cervical cytology (6.1 % vs. 16.8 %, *P* = 0.023), and recurrence (4.9 % vs. 16.8 %, *P* = 0.010) than those with a lower proportion of lactobacilli. In addition, the presence of AV was significantly associated with hrHPV infection (62.5 % vs. 43.4 %, *P* = 0.011) and recurrence (68 % vs. 46.7 %, *P* = 0.046) but not with abnormal cervical cytology (65.4 % vs. 47.0 %, *P* = 0.079). With respect to pathogen detection, no significant correlations were found between the presence of fungal spores or hyphae and the three aforementioned conditions. However, a significant association was noted between the detection of trichomonads and hrHPV infection (Table 3).

**Table 3:** Associations between postsurgical VM indices, hrHPV status, cervical cytology, and disease recurrence.

VM indices	hrHPV			Cervical cytology			Recurrence		
	Negative (n = 143)	Positive (n = 64)	P	Normal (n = 181)	Abnormal (n = 26)	P	No (n = 182)	Yes (n = 25)	P
Bacterial density									
I	16	5	0.847	21	0	0.081	19	2	0.831
II–III	81	39		105	15		104	16	
IV	46	20		55	11		59	7	

Flora diversity									
I	9	3	0.188	11	1	0.541	10	2	0.226
II–III	119	50		148	21		152	17	
IV	15	11		22	4		20	6	

Lactobacilli proportion									
<50 %	73	52	**<0.001**	104	21	**0.023**	104	21	**0.010**
≥50 %	70	12		77	5		78	4	

Leukocyte									
<10/HF	95	42	0.910	122	15	0.328	123	14	0.251
≥10/HF	48	22		59	11		59	11	

Pathogen									
Spore/hyphae	10	6	0.755	15	3	0.859	14	2	1.000
Trichomonad	0	3	**0.029**	3	0	1.000	3	0	1.000

BV									
Absence	129	52	0.072	158	23	0.865	161	20	0.231
Presence	14	12		23	3		21	5	

AV									
Absence	81	24	**0.011**	96	9	0.079	97	8	**0.046**
Presence	62	40		85	17		85	17	

pH									
<4.5	86	16	**<0.001**	97	5	**0.001**	97	5	**0.002**
≥4.5	57	48		84	21		85	20	

Leukocyte esterase									
Negative	86	30	0.076	102	14	0.810	104	12	0.388
Positive	57	34		79	12		78	13	

**Fig. 2:**
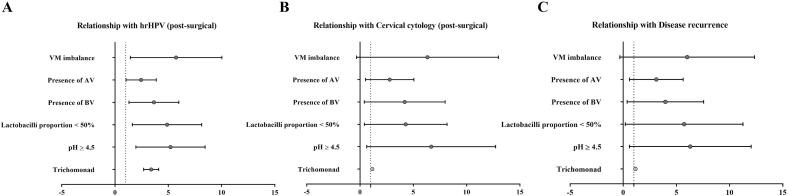
(A) Odds ratios and 95 % confidence intervals of the associations between postsurgical VM and hr-HPV infection. (B) Odds ratios and 95 % confidence intervals of the associations between postsurgical VM and cervical cytology. (C) Odds ratios and 95 % confidence intervals of the associations between postsurgical VM and disease recurrence. The bars represent 95 % confidence intervals. OR: odds ratio. The red dots signify significant positive correlations (*P* < 0.05), and the gray dots indicate nonsignificance (*P* > 0.05). (For interpretation of the references to colour in this figure legend, the reader is referred to the web version of this article.)

The impact of VM and specific indices on the risk of postsurgical hrHPV, cervical cytology, and disease recurrence was further analyzed. As shown in Table 4 and Fig. 2, VM imbalance, pH above 4.5, and a lactobacilli proportion below 50 % were identified as risk factors for postsurgical hrHPV infection, abnormal cervical cytology, and recurrence. The presence of AV was also found to be a risk factor for postsurgical hrHPV infection and recurrence but not for abnormal cervical cytology. Trichomoniasis infection was observed to be a risk factor solely for hrHPV infection.

**Table 4:** Predictions of hrHPV status, cervical cytology, and disease recurrence according to postsurgical VM indices.

Variable	hrHPV		Cervical cytology		Recurrence	
	OR [95 % CI]	P	OR [95 % CI]	P	OR [95 % CI]	P
VM imbalance	4.640 [2.085–10.460]	**<0.001**	3.994 [1.154–13.826]	**0.020**	3.789 [1.091–13.154]	**0.026**
pH≥4.5	4.530 [2.350–8.740]	**<0.001**	4.850 [1.750–13.420]	**0.001**	4.570 [1.640–12.690]	**0.002**
Lactobacilli proportion < 50 %	4.155[2.047–8.436]	**<0.001**	3.110 [1.123–8.614]	**0.023**	3.938[1.299–11.934]	**0.010**
Trichomonad	3.344 [2.711–4.126]	**0.029**	1.146 [1.088–1.208]	1.000	1.140 [1.083–1.200]	1.000
Presence of BV	2.126 [0.922–4.904]	0.072	0.896 [0.249–3.223]	0.865	1.917 [0.651–5.646]	0.231
Presence of AV	2.177 [1.190–3.986]	**0.011**	2.133 [0.904–5.307]	0.079	2.425 [0.996––5.901]	**0.046**

## 4 Discussion

Surgery plays a crucial role in the treatment of CC and CIN. The use of antibiotics and irrigation both before and after surgery may lead to presurgical VM failing to accurately represent the postsurgical condition. Despite extensive research outlining the link between VM and cervical-related diseases, substantial prospective clinical trials exploring the connection between postsurgical VM and CIN/CC are lacking. There are very few accompanying data that provide some insights (Caselli et al., 2020), making it difficult to provide direct guidance for clinical practice. Hence, we undertook this study to address these gaps.

In our study, postsurgical VM imbalance was significantly associated with hrHPV infection, abnormal cervical cytology and disease recurrence. Among the specific VM indices, a lactobacilli proportion less than 50 % was a significant risk factor for these conditions. Our findings align with those of an observational study conducted by Caselli et al. (2020). In that study, postsurgical hrHPV status was associated with postsurgical vaginal microbial types in patients who underwent surgical treatment for CIN (Caselli et al., 2020). The proportion of nonlactobacilli-dominant VM was approximately 29 % in hrHPV-positive patients, which was greater than the 10 % reported in hrHPV-negative patients; however, the difference was not statistically significant (Caselli et al., 2020). In the present study, the percentage of nonlactobacilli-dominant VMs in the hrHPV-positive group was approximately 81.3 %, which was significantly greater than the 51.0 % reported in the hrHPV-negative group (*P* < 0.001).

Trichomonads are protozoan parasites responsible for trichomoniasis, the most common nonviral sexually transmitted infection, which severely damages the reproductive system (Ibáñez-Escribano and Nogal-Ruiz, 2024). Trichomonad infection increases the risk of hrHPV infection, thereby promoting the development of cervical cancer (Hamar et al., 2023, Zhang et al., 2023). This study also confirmed that trichomonad infection is a risk factor for hrHPV infection in postsurgical CIN and CC patients. However, trichomonad infection was not observed to increase the risk of cervical cytological abnormalities or recurrence in these patients, which may be attributed to the nature of the study. This was a single-center retrospective study, and the study’s design was its main limitation, as it inherently limited the number of cases included.

A significant negative correlation between vaginal lactobacilli and hrHPV infection, as well as the development and progression of CIN/CC, has been reported previously, suggesting a potential protective role of lactobacilli in female health (Wang et al., 2019). This finding is consistent with the results of our observational study. Furthermore, various probiotics have been shown to inhibit vaginal pathogens, improve the microenvironment, and even exhibit anticancer effects *in vitro* (López-Moreno and Aguilera, 2021, Cai et al., 2023, Chung et al., 2023). Therefore, investigating whether VM intervention in patients with CIN or CC who have been surgically treated can aid in the recovery of these patients could be a meaningful and valuable research direction in the future.

The evaluation methods used for lower genital tract infections in women vary in China, including traditional cleanliness assessments commonly used in clinical practice, the VM evaluation employed in this study, and the prevalent 16S rRNA sequencing used in scientific research. Among these methods, the cleanliness assessment is the most historically utilized approach. However, this technique is limited to the examination of secretions under a low-power microscope and lacks staining microscopy and enzyme activity analysis, thus resulting in significant constraints. The 16S rRNA sequencing technique, driven by high-throughput sequencing, represents a novel method capable of comprehensive bacterial composition and quantification analysis within vaginal secretions (Wang et al., 2024). However, this method lacks direct observational outcomes, precluding visual inspection of eukaryotes such as cells, trichomonads, and fungi, as well as enzyme activity and pH data acquisition. Compared with cleanliness assessment and high-throughput sequencing, the VM assessment method utilized in this study integrates high-power microscopic observation, Gram staining, and enzyme activity analysis, presenting a more comprehensive and meticulous approach. On the basis of the results from Gram staining and oil microscopy, we can conduct quantitative Donders and Nugent scoring for the straightforward diagnosis of AV and BV. Furthermore, our analysis indicated that AV serves as a significant risk factor for postsurgical recurrence among patients (Table 4 and Fig. 2).

This study is a retrospective investigation, inevitably accompanied by certain limitations, such as selection bias. Our inclusion/exclusion criteria resulted in missing data for patients who did not adhere to regular visits because of factors such as distance from medical centers, economic status, and educational level. Additionally, the limited number of cases in this study precluded more detailed subgroup analyses, such as grouping patients who underwent complete versus incomplete cervical excision during surgery and comparing patients with carcinoma to those with dysplasia. These subgroup analyses would have been instrumental in further elucidating the mechanisms underlying the interaction between the vaginal microbiome and disease recurrence in postsurgical patients with CIN/CC.

## Funding

This study was supported by grants from the Natural Science Foundation of Anhui Province (no. 2208085MH253), the National Natural Science Foundation (no. 81702560), the Youth Fund Project of the First Affiliated Hospital of USTC (no. 2023YJQN006 and 2024YJQN010), People’s Republic of China.

## Ethics approval

Ethical approval was granted by the Ethics Committee of the First Affiliated Hospital of the University of Science and Technology of China (file record: 2022-RE-379). Because of the retrospective and anonymous nature of this study, the need for informed consent was waived by the Ethics Committee of the First Affiliated Hospital of the University of Science and Technology of China. All methods were performed in accordance with the tenets of the Declaration of Helsinki.

## CRediT authorship contribution statement

**Yan Ma:** Writing – original draft, Funding acquisition, Formal analysis, Data curation, Conceptualization. **Lijuan Wan:** Investigation, Funding acquisition. **Ruonan Li:** Writing – review & editing. **Xixi Chen:** Validation, Formal analysis. **Huiyan Wang:** Writing – review & editing, Writing – original draft, Investigation, Funding acquisition, Conceptualization.

## Declaration of competing interest

The authors declare that they have no known competing financial interests or personal relationships that could have appeared to influence the work reported in this paper.
