# Supplementary material for: Impact of postsurgical vaginal microbiome on high-risk HPV infection and recurrence risk in patients with cervical cancer and intraepithelial neoplasia: A retrospective study
Source: Gynecol Oncol Rep. 2024 Sep 11;55:101506. doi: 10.1016/j.gore.2024.101506 (PMC11416653; doi:10.1016/j.gore.2024.101506)
Supplement: Supplementary Data 1 [file mmc1.docx]

**Supplementary data**

**Supplementary Table 1.** Clinical data of the 231 women fulfilled study criteria in this study.

| ID | Age | Menopausal status | Surgical modality | Diagnosis at time of index surgery | Surgical Margin | Postsurgical vaginal microbiome | Postsurgical hrHPV | Postsurgical TCT | Recurrence |
| --- | --- | --- | --- | --- | --- | --- | --- | --- | --- |
| 1 | 40 | Pre- | LEEP | CIN2 | Negative | Balance | Negative | Normal | No |
| 2 | 24 | Pre- | LEEP | CIN3 | Negative | Imbalance | Negative | Normal | No |
| 3 | 26 | Pre- | LEEP | CIN3 | Negative | Balance | Negative | Normal | No |
| 4 | 39 | Pre- | LEEP | CIN2 | Negative | Imbalance | Negative | Normal | No |
| 5 | 31 | Pre- | LEEP | CIN2 | Negative | Imbalance | Negative | Normal | No |
| 6 | 45 | Pre- | LEEP | CIN2 | Negative | Balance | Negative | Normal | No |
| 7 | 37 | Pre- | LEEP | CIN2 | Negative | Balance | Negative | Normal | No |
| 8 | 46 | Pre- | LEEP | CIN2 | Negative | Balance | Negative | Normal | No |
| 9 | 49 | Pre- | LEEP | CIN2 | Negative | Imbalance | Negative | Normal | No |
| 10 | 51 | Pre- | LEEP | CIN1 | Negative | Imbalance | Negative | Normal | No |
| 11 | 32 | Pre- | LEEP | CIN2 | Negative | Balance | Negative | Normal | No |
| 12 | 55 | Post- | LEEP | CIN1 | Negative | Imbalance | Negative | Normal | No |
| 13 | 32 | Pre- | LEEP | CIN2 | Negative | Balance | Negative | Normal | No |
| 14 | 45 | Pre- | LEEP | CIN2 | Negative | Balance | Negative | Normal | No |
| 15 | 42 | Pre- | LEEP | CIN1 | Negative | Imbalance | Negative | Normal | No |
| 16 | 43 | Pre- | LEEP | CIN2 | Negative | Balance | Negative | Normal | No |
| 17 | 51 | Pre- | LEEP | CIN3 | Negative | Imbalance | Negative | Normal | No |
| 18 | 34 | Pre- | LEEP | CIN3 | Negative | Imbalance | Other high-risk | Normal | No |
| 19 | 42 | Pre- | CKC | CIN3 | Negative | Balance | Negative high-risk | Normal | No |
| 20 | 43 | Pre- | LEEP | CIN2 | Negative | Imbalance | Other high-risk | ASCUS | No |
| 21 | 30 | Pre- | LEEP | CIN3 | Negative | Balance | Negativee | Normal | No |
| 22 | 45 | Pre- | LEEP | CIN3 | Negative | Imbalance | Other high-risk | ASCUS | No |
| 23 | 47 | Pre- | LEEP | CIN3 | Negative | Imbalance | 16 | Normal | No |
| 24 | 38 | Pre- | LEEP | CIN3 | Negative | Imbalance | 18 | Normal | No |
| 25 | 47 | Pre- | LEEP | CIN3 | Negative | Imbalance | Other high-risk | Normal | No |
| 26 | 56 | Post- | LEEP | CIN2 | Negative | Imbalance | Other high-risk | Normal | No |
| 27 | 38 | Pre- | LEEP | CIN2 | Negative | Balance | Other high-risk | Normal | No |
| 28 | 52 | Post- | LEEP | CIN2 | Negative | Imbalance | Other high-risk | LSIL | Yes (CIN1) |
| 29 | 35 | Pre- | CKC | CIN3 | Negative | Balance | Negative | Normal | No |
| 30 | 59 | Post- | LEEP | CIN1 | Negative | Imbalance | Other high-risk | Normal | Yes (CIN1) |
| 31 | 46 | Pre- | LEEP | CIN2 | Negative | Imbalance | Other high-risk | Normal | No |
| 32 | 57 | Post- | LEEP | CIN1 | Negative | Imbalance | 18 | Normal | Yes (CIN1) |
| 33 | 30 | Pre- | LEEP | CIN2 | Negative | Imbalance | Other high-risk | Normal | No |
| 34 | 37 | Pre- | CKC | CIN3 | Negative | Imbalance | Negative | Normal | No |
| 35 | 53 | Post- | CKC | CIN3 | Negative | Imbalance | Negative | Normal | No |
| 36 | 41 | Pre- | CKC | CIN3 | Negative | Balance | Negative | Normal | No |
| 37 | 42 | Pre- | CKC | CIN3 | Negative | Imbalance | Negative | Normal | No |
| 38 | 50 | Pre- | CKC | CIN3 | Negative | Balance | Negative | Normal | No |
| 39 | 60 | Post- | CKC | CIN3 | Negative | Balance | Negative | Normal | No |
| 40 | 46 | Pre- | CKC | CIN3 | Negative | Balance | Negative | Normal | No |
| 41 | 47 | Post- | CKC | CIN3 | Negative | Imbalance | Negative | Normal | No |
| 42 | 36 | Pre- | CKC | CIN2 | Negative | Imbalance | Negative | Normal | No |
| 43 | 34 | Pre- | CKC | CIN3 | Negative | Imbalance | Negative | Normal | No |
| 44 | 45 | Pre- | CKC | CIN3 | Negative | Imbalance | Negative | Normal | No |
| 45 | 36 | Pre- | CKC | CIN3 | Negative | Balance | Negative | Normal | No |
| 46 | 49 | Pre- | CKC | CIN3 | Negative | Imbalance | Negative | Normal | No |
| 47 | 49 | Pre- | CKC | CIN2 | Negative | Balance | Negative | Normal | No |
| 48 | 55 | Post- | CKC | CIN3 | Negative | Imbalance | Negative | Normal | No |
| 49 | 52 | Post- | CKC | CIN3 | Negative | Balance | Negative | Normal | No |
| 50 | 45 | Pre- | CKC | CIN3 | Negative | Balance | Negative | Normal | No |
| 51 | 36 | Pre- | CKC | CIN3 | Negative | Balance | Negative | Normal | No |
| 52 | 38 | Pre- | CKC | CIN3 | Negative | Imbalance | Negative | Normal | No |
| 53 | 31 | Pre- | CKC | CIN3 | Negative | Balance | Negative | Normal | No |
| 54 | 34 | Pre- | CKC | CIN3 | Negative | Balance | Negative | Normal | No |
| 55 | 45 | Pre- | CKC | CIN3 | Negative | Imbalance | Negative | Normal | No |
| 56 | 33 | Pre- | CKC | CIN3 | Negative | Imbalance | Negative | Normal | No |
| 57 | 54 | Post- | CKC | CIN2 | Negative | Imbalance | Negative | Normal | No |
| 58 | 27 | Pre- | CKC | CIN2 | Negative | Imbalance | Negative | Normal | No |
| 59 | 35 | Pre- | CKC | CIN3 | Negative | Balance | Negative | Normal | No |
| 60 | 42 | Pre- | CKC | CIN3 | Negative | Balance | Negative | Normal | No |
| 61 | 47 | Pre- | CKC | CIN3 | Negative | Balance | Negative | Normal | No |
| 62 | 35 | Pre- | CKC | CIN3 | Negative | Balance | Negative | Normal | No |
| 63 | 50 | Pre- | CKC | CIN2 | Negative | Balance | Negative | Normal | No |
| 64 | 57 | Post- | CKC | CIN3 | Negative | Imbalance | Negative | Normal | No |
| 65 | 40 | Pre- | CKC | CIN3 | Negative | Imbalance | Negative | Normal | No |
| 66 | 56 | Post- | CKC | CIN3 | Negative | Imbalance | Negative | Normal | No |
| 67 | 56 | Post- | CKC | CIN3 | Negative | Imbalance | Negative | Normal | No |
| 68 | 39 | Pre- | CKC | CIN3 | Negative | Imbalance | Negative | Normal | No |
| 69 | 51 | Pre- | CKC | CIN3 | Negative | Balance | Negative | Normal | No |
| 70 | 51 | Pre- | CKC | CIN3 | Negative | Imbalance | Negative | Normal | No |
| 71 | 46 | Pre- | CKC | CIN3 | Negative | Imbalance | Negative | Normal | No |
| 72 | 34 | Pre- | CKC | CIN3 | Negative | Imbalance | Negative | Normal | No |
| 73 | 47 | Pre- | CKC | CIN2 | Negative | Balance | Negative | Normal | No |
| 74 | 28 | Pre- | CKC | CIN3 | Negative | Imbalance | Negative | Normal | No |
| 75 | 43 | Pre- | CKC | CIN3 | Negative | Balance | Negative | Normal | No |
| 76 | 36 | Pre- | CKC | CIN3 | Negative | Imbalance | Negative | AUSCS | Yes (CIN1) |
| 77 | 30 | Pre- | CKC | CIN3 | Negative | Imbalance | Negative | AUSCS | No |
| 78 | 56 | Post- | CKC | CIN3 | Negative | Imbalance | Other high-risk | Normal | Yes (CIN1) |
| 79 | 48 | Pre- | CKC | CIN3 | Negative | Balance | Other high-risk | Normal | No |
| 80 | 35 | Pre- | CKC | CIN3 | Negative | Imbalance | 16 | Normal | No |
| 81 | 30 | Pre- | CKC | CIN3 | Negative | Imbalance | Other high-risk | Normal | No |
| 82 | 52 | Post- | CKC | CIN3 | Negative | Imbalance | Other high-risk | Normal | No |
| 83 | 45 | Pre- | CKC | CIN3 | Negative | Imbalance | Other high-risk | Normal | No |
| 84 | 38 | Pre- | CKC | CIN2 | Negative | Imbalance | Other high-risk | Normal | No |
| 85 | 42 | Pre- | CKC | CIN3 | Negative | Imbalance | Other high-risk | Normal | No |
| 86 | 44 | Pre- | CKC | CIN3 | Negative | Imbalance | Other high-risk | Normal | No |
| 87 | 27 | Pre- | CKC | CIN3 | Negative | Imbalance | Other high-risk | Normal | No |
| 88 | 34 | Pre- | CKC | CIN3 | Negative | Imbalance | Other high-risk | Normal | Yes (CIN1) |
| 89 | 51 | Pre- | CKC | CIN2 | Negative | Imbalance | 18 | ASCUS | No |
| 90 | 57 | Post- | CKC | CIN2 | Negative | Imbalance | 16 | Normal | No |
| 91 | 44 | Pre- | CKC | CIN3 | Negative | Imbalance | Other high-risk | Normal | No |
| 92 | 45 | Pre- | CKC | CIN3 | Negative | Imbalance | Other high-risk | Normal | No |
| 93 | 42 | Pre- | CKC | CIN3 | Negative | Balance | Other high-risk | ASCUS | Yes (CIN1) |
| 94 | 44 | Pre- | CKC | CIN3 | Negative | Imbalance | Other high-risk | Normal | Yes (CIN1) |
| 95 | 55 | Post- | CKC | CIN3 | Negative | Imbalance | Other high-risk | Normal | No |
| 96 | 47 | Pre- | CKC | CIN3 | Negative | Imbalance | Other high-risk | ASCUS | Yes (CIN1) |
| 97 | 36 | Pre- | CKC | CIN3 | Negative | Imbalance | Other high-risk | ASCUS | Yes (CIN1) |
| 98 | 40 | Pre- | CKC | CIN3 | Negative | Imbalance | Other high-risk | Normal | No |
| 99 | 45 | Pre- | CKC | CIN2 | Negative | Imbalance | Other high-risk | Normal | No |
| 100 | 43 | Pre- | CKC | CIN3 | Negative | Balance | Other high-risk | Normal | No |
| 101 | 44 | Pre- | [TLH](javascript:;) | CIN3 | Negative | Imbalance | Negative | Normal | No |
| 102 | 51 | Post- | TLH | CIN2 | Negative | Imbalance | Negative | Normal | No |
| 103 | 57 | Post- | TLH | CIN2 | Negative | Balance | Negative | Normal | No |
| 104 | 40 | Pre- | TLH | CIN3 | Negative | Balance | Negative | Normal | No |
| 105 | 73 | Pre- | TLH | CIN3 | Negative | Imbalance | Negative | Normal | No |
| 106 | 32 | Pre- | CKC | CIN3 | Negative | Balance | Negative | Normal | No |
| 107 | 52 | Post- | TLH | CIN3 | Negative | Imbalance | Negative | ASCUS | No |
| 108 | 49 | Post- | TLH | CIN3 | Negative | Imbalance | Negative | ASCUS | Yes (CIN1) |
| 109 | 57 | Post- | TLH | CIN3 | Negative | Imbalance | Other high-risk | Normal | No |
| 110 | 51 | Pre- | TLH | CIN3 | Negative | Imbalance | Other high-risk | Normal | No |
| 111 | 53 | Post- | TLH | CIN3 | Negative | Imbalance | Other high-risk | Normal | No |
| 112 | 60 | Post- | TLH | CIN3 | Negative | Imbalance | 16 | Normal | Yes (CIN2) |
| 113 | 52 | Post- | TLH | SCC | Negative | Imbalance | Negative | Normal | No |
| 114 | 56 | Post- | TLH | AC | Negative | Imbalance | Negative | Normal | No |
| 115 | 51 | Post- | TLH | SCC | Negative | Imbalance | Negative | LSIL | Yes (CIN1) |
| 116 | 54 | Post- | TLH | SCC | Negative | Imbalance | Negative | ASCUS | Yes (CIN1) |
| 117 | 49 | Pre- | RH | SCC | Negative | Imbalance | Negative | Normal | No |
| 118 | 53 | Post- | RH | SCC | Negative | Imbalance | Negative | Normal | No |
| 119 | 33 | Pre- | RH | SCC | Negative | Balance | Negative | Normal | No |
| 120 | 45 | Pre- | RH | SCC | Negative | Imbalance | Negative | Normal | No |
| 121 | 41 | Pre- | RH | SCC | Negative | Imbalance | Negative | Normal | No |
| 122 | 45 | Pre- | RH | SCC | Negative | Balance | Negative | Normal | No |
| 123 | 31 | Pre- | RH | SCC | Negative | Balance | Negative | Normal | No |
| 124 | 47 | Pre- | RH | SCC | Negative | Balance | Negative | Normal | No |
| 125 | 36 | Pre- | RH | SCC | Negative | Imbalance | Negative | Normal | No |
| 126 | 48 | Pre- | RH | SCC | Negative | Imbalance | Negative | Normal | No |
| 127 | 63 | Post- | RH | SCC | Negative | Balance | Negative | Normal | No |
| 128 | 49 | Pre- | RH | ASC | Negative | Imbalance | Negative | Normal | No |
| 129 | 46 | Pre- | RH | AC | Negative | Balance | Negative | Normal | No |
| 130 | 49 | Pre- | RH | SCC | Negative | Balance | Negative | Normal | No |
| 131 | 35 | Pre- | RH | SCC | Negative | Imbalance | Negative | Normal | No |
| 132 | 49 | Pre- | RH | AC | Negative | Imbalance | Negative | Normal | No |
| 133 | 46 | Pre- | RH | SCC | Negative | Imbalance | Negative | Normal | No |
| 134 | 50 | Post- | RH | SCC | Negative | Balance | Negative | Normal | No |
| 135 | 46 | Pre- | RH | SCC | Negative | Imbalance | Negative | Normal | No |
| 136 | 41 | Pre- | RH | SCC | Negative | Imbalance | Negative | Normal | No |
| 137 | 46 | Pre- | RH | SCC | Negative | Imbalance | Negative | Normal | No |
| 138 | 58 | Post- | RH | SCC | Negative | Balance | Negative | Normal | No |
| 139 | 39 | Pre- | RH | SCC | Negative | Imbalance | Negative | ASCUS | No |
| 140 | 61 | Post- | RH | SCC | Negative | Imbalance | Negative | ASCUS | No |
| 141 | 59 | Post- | TLH | SCC | Negative | Imbalance | 16 | Normal | Yes (CIN1) |
| 142 | 61 | Post- | RH | SCC | Negative | Imbalance | Other high-risk | Normal | Yes (CIN1) |
| 143 | 57 | Post- | RH | AC | Negative | Imbalance | Other high-risk | Normal | No |
| 144 | 39 | Pre- | CKC | AC | Negative | Balance | \| Negative  Normal \| \| --- \|   Other high-risk | Normal | No |
| 145 | 49 | Pre- | RH | SCC | Negative | Imbalance | 16 | ASCUS | Yes (CIN1) |
| 146 | 48 | Pre- | RH | SCC | Negative | Imbalance | Other high-risk | ASCUS | Yes (CIN1) |
| 147 | 32 | Pre- | RH | SCC | Negative | Imbalance | Other high-risk | Normal | No |
| 148 | 49 | Pre- | RH | SCC | Negative | Imbalance | Other high-risk | Normal | No |
| 149 | 36 | Pre- | RH | SCC | Negative | Imbalance | Other high-risk | ASCUS | No |
| 150 | 45 | Pre- | RH | SCC | Negative | Imbalance | Other high-risk | Normal | Yes (CIN1) |
| 151 | 57 | Post- | RH | SCC | Negative | Imbalance | Other high-risk | Normal | No |
| 152 | 45 | Post- | LEEP | CIN1 | Negative | Balance | Negative | Normal | No |
| 153 | 40 | Pre- | CKC | CIN2 | Negative | Imbalance | Negative | Normal | No |
| 154 | 42 | Pre- | CKC | CIN3 | Negative | Imbalance | Negative | Normal | No |
| 155 | 51 | Post- | RH | SCC | Negative | Imbalance | Negative | LSIL | No |
| 156 | 56 | Post- | RH | SCC | Negative | Imbalance | Negative | Normal | No |
| 157 | 56 | Post- | CKC | CIN3 | Negative | Imbalance | Negative | Normal | No |
| 158 | 39 | Post- | CKC | CIN3 | Negative | Imbalance | Negative | Normal | No |
| 159 | 51 | Pre- | CKC | CIN3 | Negative | Balance | Negative | Normal | No |
| 160 | 45 | Pre- | RH | SCC | Negative | Imbalance | Negative | Normal | No |
| 161 | 52 | Post- | RH | SCC | Negative | Imbalance | Negative | Normal | No |
| 162 | 55 | Post- | RH | SCC | Negative | Balance | Negative | Normal | No |
| 163 | 51 | Pre- | RH | SCC | Negative | Imbalance | Negative | LSIL | No |
| 164 | 46 | Pre- | LEEP | CIN1 | Positive | Imbalance | Negative | Normal | No |
| 165 | 21 | Pre- | LEEP | CIN1 | Positive | Imbalance | Negative | ASCUS | No |
| 166 | 53 | Pre- | CKC | CIN3 | Positive | Balance | Negative | Normal | No |
| 167 | 47 | Pre- | CKC | CIN3 | Positive | Balance | Negative | Normal | No |
| 168 | 38 | Pre- | CKC | CIN3 | Positive | Balance | Negative | Normal | No |
| 169 | 44 | Pre- | CKC | CIN3 | Positive | Imbalance | Negative | Normal | No |
| 170 | 44 | Pre- | CKC | CIN3 | Positive | Imbalance | Negative | Normal | No |
| 171 | 45 | Pre- | TLH | CIN3 | Positive | Imbalance | Negative | Normal | No |
| 172 | 38 | Pre- | LEEP | CIN2 | Positive | Imbalance | Other high-risk | ASCUS | No |
| 173 | 34 | Pre- | CKC | CIN3 | Positive | Imbalance | Other high-risk | Normal | No |
| 174 | 51 | Pre- | RH | SCC | Negative | Imbalance | Negative | Normal | No |
| 175 | 41 | Pre- | TLH | SCC | Negative | Imbalance | Negative | Normal | No |
| 176 | 55 | Post- | RH | SCC | Negative | Imbalance | Negative | Normal | No |
| 177 | 47 | Pre- | LEEP | CIN2 | Negative | Imbalance | Negative | Normal | No |
| 178 | 26 | Pre- | CKC | CIN3 | Negative | Balance | Negative | Normal | No |
| 179 | 57 | Post- | TLH | SCC | Negative | Imbalance | Negative | Normal | Yes (CIN1) |
| 180 | 40 | Pre- | CKC | CIN3 | Negative | Imbalance | Negative | Normal | No |
| 181 | 30 | Pre- | CKC | CIN3 | Negative | Balance | Negative | Normal | No |
| 182 | 45 | Pre- | RH | SCC | Negative | Imbalance | Negative | Normal | No |
| 183 | 55 | Post- | RH | SCC | Negative | Imbalance | Negative | Normal | No |
| 184 | 50 | Pre- | CKC | CIN3 | Negative | Balance | Negative | Normal | No |
| 185 | 51 | Pre- | TLH | CIN1 | Negative | Imbalance | Other high-risk | Normal | No |
| 186 | 51 | Pre- | RH | SCC | Negative | Balance | Negative | Normal | No |
| 187 | 46 | Pre- | CKC | CIN3 | Negative | Balance | Other high-risk | Normal | No |
| 188 | 47 | Pre- | LEEP | CIN3 | Negative | Balance | Negative | Normal | No |
| 189 | 48 | Pre- | LEEP | CIN2 | Negative | Imbalance | Negative | Normal | No |
| 190 | 70 | Post- | LEEP | CIN3 | Negative | Imbalance | Negative | Normal | No |
| 191 | 54 | Post- | CKC | CIN3 | Negative | Imbalance | Other high-risk | LSIL | No |
| 192 | 50 | Post- | CKC | CIN3 | Negative | Balance | Negative | Normal | No |
| 193 | 38 | Pre- | CKC | CIN3 | Negative | Imbalance | Negative | Normal | No |
| 194 | 38 | Pre- | CKC | CIN3 | Negative | Imbalance | Negative | Normal | No |
| 195 | 65 | Post- | LEEP | CIN1 | Negative | Imbalance | Negative | Normal | No |
| 196 | 28 | Pre- | LEEP | CIN2 | Negative | Imbalance | Negative | Normal | No |
| 197 | 50 | Post- | CKC | CIN3 | Negative | Imbalance | Negative | Normal | No |
| 198 | 37 | Pre- | CKC | CIN3 | Negative | Balance | Negative | Normal | No |
| 199 | 46 | Pre- | CKC | CIN3 | Negative | Imbalance | Negative | Normal | No |
| 200 | 38 | Pre- | CKC | CIN3 | Negative | Imbalance | Negative | Normal | No |
| 201 | 56 | Post- | CKC | CIN3 | Negative | Imbalance | Negative | Normal | No |
| 202 | 41 | Pre- | CKC | CIN3 | Negative | Balance | Negative | Normal | No |
| 203 | 51 | Pre- | LEEP | CIN1 | Negative | Imbalance | Other high-risk | LSIL | No |
| 204 | 46 | Pre- | LEEP | CIN3 | Negative | Imbalance | Other high-risk | Normal | No |
| 205 | 39 | Pre- | LEEP | CIN1 | Negative | Imbalance | Other high-risk | ASCUS | No |
| 206 | 33 | Pre- | LEEP | CIN1 | Negative | Balance | Other high-risk | LSIL | No |
| 207 | 58 | Post- | CKC | CIN1 | Negative | Balance | Other high-risk | LSIL | Yes (CIN1) |
| 208 | 54 | Post- | CKC | CIN2 | Negative | Balance | Negative | Normal | No |
| 209 | 35 | Pre- | LEEP | CIN2 | Negative | Imbalance | Negative | Normal | No |
| 210 | 47 | Pre- | CKC | CIN3 | Negative | Balance | Negative | Normal | No |
| 211 | 36 | Pre- | CKC | CIN2 | Negative | Balance | 16 | Normal | Yes (CIN3) |
| 212 | 54 | Post- | CKC | CIN3 | Negative | Imbalance | Negative | Normal | No |
| 213 | 49 | Pre- | CKC | CIN3 | Negative | Balance | Negative | Normal | No |
| 214 | 44 | Pre- | CKC | CIN3 | Negative | Imbalance | 16 | ASCUS | Yes (CIN2) |
| 215 | 34 | Pre- | CKC | CIN3 | Negative | Imbalance | 16 | ASCUS | Yes (CIN3) |
| 216 | 50 | Post- | CKC | CIN2 | Negative | Imbalance | Negative | Normal | No |
| 217 | 50 | Post- | CKC | CIN3 | Negative | Imbalance | Negative | Normal | No |
| 218 | 51 | Post- | LEEP | CIN2 | Negative | Imbalance | Negative | Normal | No |
| 219 | 27 | Pre- | CKC | CIN2 | Negative | Imbalance | Negative | Normal | No |
| 220 | 37 | Pre- | CKC | CIN3 | Negative | Imbalance | Negative | Normal | No |
| 221 | 36 | Pre- | LEEP | CIN2 | Negative | Balance | Negative | Normal | No |
| 222 | 35 | Pre- | CKC | CIN3 | Negative | Balance | Negative | Normal | No |
| 223 | 49 | Pre- | CKC | CIN3 | Negative | Imbalance | Negative | Normal | No |
| 224 | 32 | Pre- | LEEP | CIN2 | Negative | Imbalance | Other high-risk | Normal | No |
| 225 | 43 | Pre- | LEEP | CIN3 | Negative | Imbalance | Negative | Normal | No |
| 226 | 50 | Pre- | CKC | CIN3 | Negative | Imbalance | Negative | Normal | No |
| 227 | 53 | Post- | TLH | CIN3 | Negative | Imbalance | Negative | Normal | No |
| 228 | 41 | Pre- | TLH | CIN3 | Negative | Balance | Negative | Normal | No |
| 229 | 31 | Pre- | LEEP | CIN3 | Negative | Imbalance | Negative | Normal | No |
| 230 | 43 | Pre- | CKC | CIN3 | Negative | Balance | Negative | Normal | No |
| 231 | 52 | Post- | TLH | CIN3 | Negative | Imbalance | Other high-risk | ASCUS | Yes (CIN1) |

**Abbreviations:** ASCUS, atypical squamous cells of undetermined significance; hrHPV, high risk human papillomavirus; LSIL, low-grade squamous intraepithelial lesion; HSIL, high-grade squamous intraepithelial lesion; CIN, cervical intraepithelial neoplasia; SCC, squamous-cell carcinoma; AC, cervical adenocarcinoma; ASC, cervical adenosquamous cell carcinoma; LEEP, loop electrosurgical excision procedure; CKC, cold knife conization; TLH, total hysterectomy; RH, radical total hysterectomy.
